# Segmental resection of the duodenum for gastrointestinal stromal tumor (GIST)

**DOI:** 10.1186/1477-7819-6-105

**Published:** 2008-09-30

**Authors:** Rudolf Mennigen, Heiner H Wolters, Bernd Schulte, Friedrich W Pelster

**Affiliations:** 1 Department of General and Visceral Surgery, Muenster University, Muenster, Germany; 2 Department of Pathology, Muenster University, Muenster, Germany

## Abstract

**Background:**

Gastrointestinal stromal tumors (GIST) are the most frequent mesenchymal tumors of the gastrointestinal tract. The biological appearance of these tumors reaches from small lesions with benign appearance to aggressive sarcomas. Only 3–5% of GISTs are localized in the duodenum. There is a controversy, if duodenal GISTs should be treated by a duodenopancreatectomy or by a limited resection of the duodenum.

**Case presentation:**

A 29-year-old man presented with an acute upper gastrointestinal bleeding from a submucosal tumor located in the proximal part III of the duodenum, 3 cm distal of the papilla of Vater. After an emergency laparotomy with ligation of tumor-feeding vessels in a primary hospital, definitive surgical therapy was performed by partial resection of the duodenum with a duodenojejunostomy. Histology revealed a GIST with a diameter of 2.5 cm and <5 mitoses/50 high power fields, indicating a low risk of malignancy. Therefore no adjuvant therapy with Imatinib was initiated.

**Conclusion:**

GISTs of the duodenum are a rare cause of upper gastrointestinal bleeding. Partial resection of the duodenum is a warranted alternative to a duodenopancreatectomy, as this procedure has a lower operative morbidity, while providing comparable oncological results.

## Background

Gastrointestinal stromal tumor (GIST) is a recently recognized tumor entity. Many tumors formerly classified as smooth muscle tumors (leiomyomas, leiomyosarcomas, and leiomyoblastomas) are now redefined as GIST [1,2]. Using the newly defined diagnostic criteria, GISTs are the most common mesenchymal tumors of the gastrointestinal tract. GISTs can be recognized by their morphology (spindle cell or epithelioid), positivity for CD117 (about 95%) and to some extent their positivity for CD34 (about 70%) [1-3].

GISTs can be localized anywhere in the gastrointestinal tract, stomach (40–60%) and small intestine (30–40%) being the most common locations [1,4]. Only 3–5% of GISTs occur in the duodenum [1].

GISTs show a wide range of biological appearance, from small incidentally found tumors with benign appearance to aggressive sarcomas. As all GISTs have a malignancy potential, even though they may appear benign both macro- and microscopically, for clinical purposes the risk of recurrence and metastases is estimated by evaluating the tumor diameter and the mitotic ratio [5]. Due to this potential, GISTs should always be treated.

Surgery is the mainstay in the therapy of localized GISTs. An en-bloc resection is recommended whenever feasible. Especially for GISTs localized in the duodenum, there is a controversy about the optimal surgical treatment. Some argue that a duodenopancreatectomy provides better oncological control, others support the selective use of a limited resection of the duodenum in order to minimize operative morbidity and mortality [6].

We herein report the case of a patient with a GIST of the duodenum, located 3 cm distal to the papilla of Vater, who was successfully treated by a partial resection of the duodenum.

## Case presentation

A 29-year-old man with no history of preexisting diseases presented with acute upper gastrointestinal bleeding in a local primary hospital. Endoscopy revealed a submucosal tumor located shortly distal of the papilla of Vater, bulging under the mucosa and forming a partly intraluminal mass. The mucosa showed a central ulceration, this being the origin of the massive bleeding. Because of persistent bleeding that could not be controlled by endoscopic interventional treatment, an emergency laparotomy was performed at the local primary hospital. At laparotomy, an encapsulated mass originating from the duodenal wall at the proximal third portion of the duodenum was identified, reaching to the pancreatic head. A ligation of tumor-feeding vessels was successfully performed to control the bleeding. After the emergency treatment, the patient was referred to our university hospital for further therapy.

**Figure 1:**
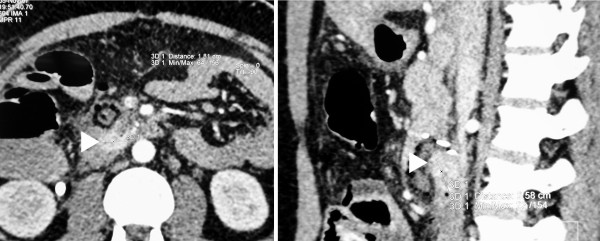
**Computed tomography**. The computed tomography showed an enhancing tumor of the duodenal wall (arrowheads) with a maximal diameter of 2.5 cm. No metastases were visualized.

Computed tomography visualized the tumor of the duodenum with a diameter of 1.8 × 2.3 × 2.5 cm (Figure 1). The scan showed no metastases. 2 days after the initial emergency surgery, the patient underwent definitive surgery at our institution. No recurrent bleeding occurred since the emergency ligation of tumor-feeding vessels.

**Figure 2:**
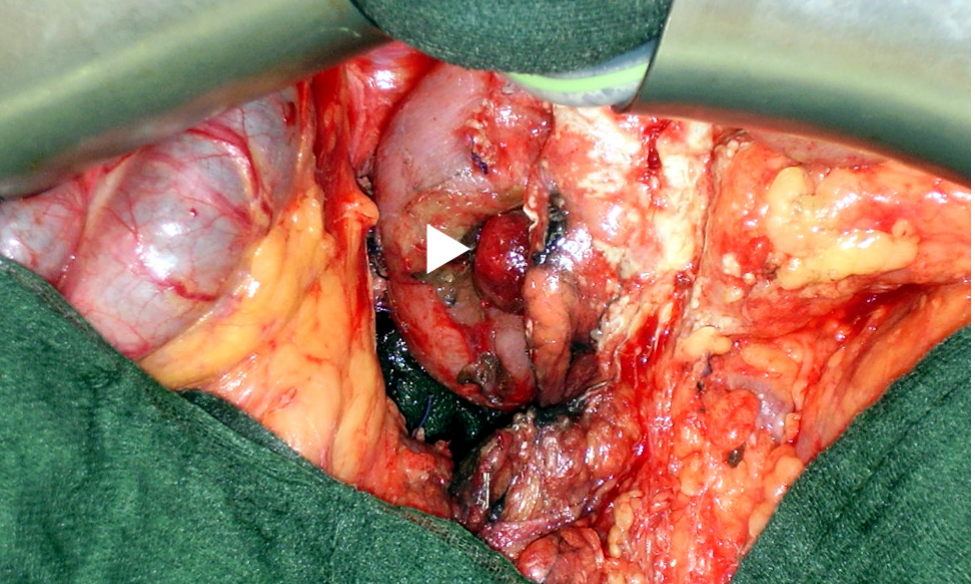
**Operative view**. Exophytic tumor of the proximal third part of the duodenum (arrowhead).

**Figure 3:**
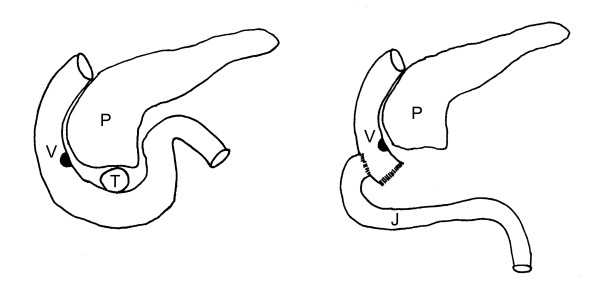
**Operative technique**. Reconstruction by a lateroterminal duodenojejunostomy at the level of the ampulla of Vater. P: pancreas, V: ampulla of Vater, T: tumor, J: jejunum.

A relaparotomy was performed. The tumor was located in the proximal third portion of the duodenum, 3 cm distal of the ampulla of Vater (Figure 2). It originated from the duodenal wall and protruded as a roundly shaped mass into the near of the pancreatic head. No infiltration of the pancreas or other adjacent organs was found, there were no suspicious lymph nodes. There were no signs of duodenal ischemia related to the haemostatic vessel ligation done at the first emergency operation. The tumor was treated by a limited resection of the distal second, third and fourth part of the duodenum, the proximal resection margin was located just distal of the ampulla of Vater. The bowel continuity was reconstructed by a latero-terminal duodenojejunostomy (Figure 3) located opposite to the ampulla of Vater in order not to induce a stricture of the papilla.

The postoperative course was uneventful and the patient was discharged on postoperative day 13.

**Figure 4:**
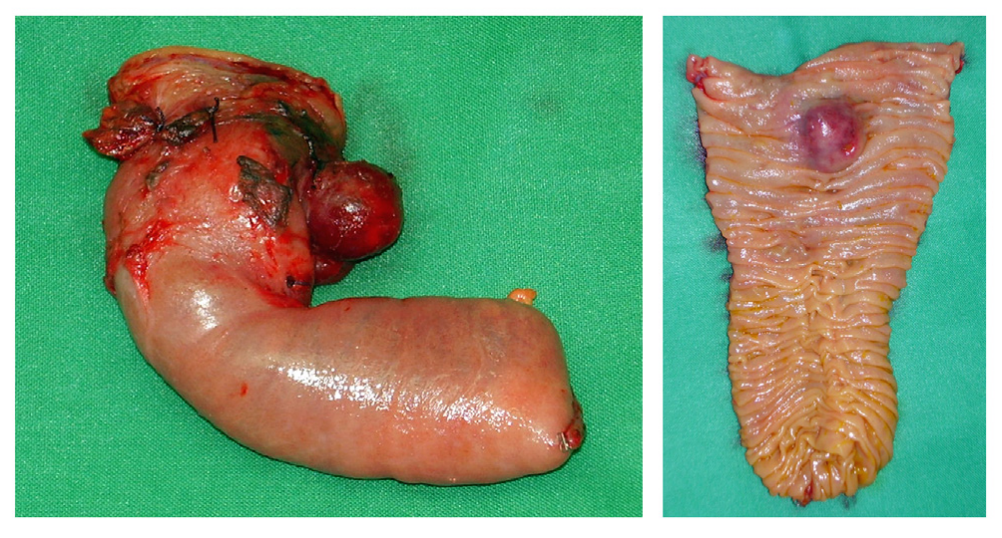
**Macroscopic appearance of the resected specimen**. The distance to the proximal resection margin was 0.5 cm. A great portion of the tumor was located subserosal. The duodenal mucosa was bulged by the sumucosal tumor.

**Figure 5:**
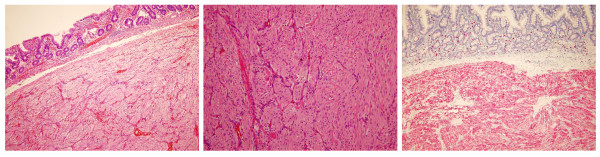
**Histology**. Microscopic appearance of the GIST located in the submucosa of the duodenum (left; H&E, original magnification ×10). It consists of small spindle-like tumor cells (middle; H&E, original magnification ×20) with CD117 – positivity (right; original magnification ×10).

In the opened specimen, the tumor diameter was 2.5 cm (Figure 4). The distance to the proximal resection margin was 0.5 cm; overall length of the duodenal segment was 9 cm. Histology revealed a GIST with a typical spindle cell pattern of the tumor cells (Figure 5). There was focal necrosis. The main tumor mass was located subserosal. The tumor had a thin fibrous capsule, and it reached the muscularis mucosae, without penetrating it. There was a regular duodenal mucosa covering the tumor. Immunohistochemistry showed a strong positivity for KIT (CD117) and CD34, while desmin and smooth muscle actin were negative (Figure 5). Mitotic activity was < 5/50 high power fields. No formal lymph node dissection had been performed, and as expected no lymph nodes were detected in the resected specimen.

As tumor diameter and low proliferative activity indicated a low risk of malignancy and recurrence, there was no indication for an adjuvant therapy with Imatinib, a tyrosine kinase inhibitor.

## Discussion

Mazur and Clark first used the term "gastrointestinal stromal tumor" in 1983 to describe gastrointestinal mesenchymal tumors that neither showed immunohistochemical and ultrastructural characteristics of neuronal Schwann cells nor of smooth muscle cells [1,7]. GISTs are believed to originate from the interstitial cells of Cajal, which are intestinal pacemaker cells or mesenchymal stem cells [8]. The origin from multipotential mesenchymal stem cells explains that both myogenic and neurogenic features may be present. GISTs remained rarely diagnosed until the late 90 ies. Nowadays, GISTs represent the most common mesenchymal tumor entity of the gastrointestinal tract.

A typical feature of virtually all GISTs is a positivity at immunohistochemistry for the KIT protein (CD117), a transmembrane receptor linked to an intracytoplasmatic tyrosine kinase [9]. In GIST, gain-of function mutations of the c-kit gene are present in about 80% [3], whereas a subset of GISTs harbors mutations in platelet-derived growth factor receptor alpha (PDGFRA, about 5%), a CD117 related tyrosine-kinase receptor [10]. Further typical findings are the positivity for vimentin (nearly all GISTs) and CD34 (50–70%) [1,2]. Staining for smooth muscle actin (SMA) may be positive (30–40%), while desmin (intermediate filament typical for muscle) and S-100 (a neural cell marker) usually are negative [1,2]. GISTs grow expansively and are often covered by a pseudocapsule.

The epidemiology of GISTs is not completely known. GIST is an infrequent neoplasm with an annual incidence of 12.7 per million in the Netherlands, and 6.8 per million in the U.S. [4,11]. GISTs can be located anywhere in the GI-tract. Most common sites are stomach (40–60%) and small intestine (30–40%) [1,4]. The mean age of patients with GIST is 53 years. Only about 5% of GIST patients are younger than 30 years [2]. Therefore, the age of our patient is quite untypical for the diagnosis of duodenal GIST.

GISTs of the duodenum make up only 4.5% of all GISTs [12] and therefore represent a rare tumor entity. In 2003, Miettinen published a clinicopathologic study on 156 duodenal GISTs giving insight into tumor characteristics and natural history [2]. Further data on duodenal GIST are mainly derived from smaller series or case reports [13-26].

Duodenal GISTs are mainly located in the second portion of the duodenum (42/156) [2]. The tumors are frequently located in close relationship to the ampulla of Vater, this determining surgical treatment strategies. In the case presented here, the tumor was located 3 cm distal of the papilla.

Most duodenal GISTs present with GI bleeding usually associated with melena, occasionally with massive acute bleeding like in our patient [2]. Other symptoms like abdominal pain, early satiety, bloating, or obstructive jaundice due to involvement of the papilla of Vater were not present in our patient. Endoscopic detection of duodenal GISTs is easily possible in case of a visual endoluminal mass. This was the case in our patient. The inner part of the tumor reached the muscularis mucosae without penetrating it, forming a partly intraluminal mass covered by mucosa. However, the main tumor was located subserosal, having a exophytic spheric part protruding from the outer duodenal wall. This is the most common type of involvement of duodenal layers by duodenal GIST (58/156) [2]. As the mucosa usually is not involved, especially smaller intra- or extramural tumors (29/156) [2] may be undetectable by endoscopy alone. Endoscopic ultrasound can be used to visualize these submucosal tumors. If endoscopy shows no abnormalities, extramural tumor masses located close to the pancreas may mimic a pancreatic head tumor [13,21] eventually leading to a duodenopancreatectomy. The extraluminal portion of the GIST was located close to the pancreatic head in our patient, too. A CT scan can visualize the duodenal tumor and possible metastases (Fig. 1).

In our patient, acute upper GI bleeding that could not be controlled by endoscopy led to an emergency operation with ligation of tumor-feeding vessels at a primary hospital before the patient was referred to our institution. Kurihara reported transarterial embolization to be a possible alternative to control acute bleeding from duodenal GIST [25]. This procedure should be considered in case of acute bleeding from duodenal GIST, if angiography is available within reasonable time.

The biological appearance of GISTs shows a great variance. About 30% of GISTs lead to local recurrence and metastases [2,27]. The overall 5-year survival rate for GIST patients is about 45% in the USA [11]. Fletcher established a risk stratification based upon tumor diameter and mitotic activity [27]. The tumor presented in this case belongs to the category determined by size between 2–5 cm and a mitotic count <5/50 high power fields, which is classified as "low risk". As we performed a complete resection of the GIST, this indicates a good prognosis for our patient. But even in this "low risk" group, 8% of patients develop recurrence, metastasis, or die of tumor [2]. Therefore a scheduled follow up of the patient is mandatory, and should be continued for many years, maybe lifelong, due to the slow growth of GISTs.

Surgery is the therapy of choice for localized GIST. GISTs can give rise to metastases in the peritoneal cavity and in the liver, and in a lesser frequency, in lung, and bones. In contrast, a lymph nodal spread is uncommon, and a formal lymph node dissection has no proven value [1,2,28]. In our patient, no suspicious peritumoral lymph nodes were present. Therefore, in order to minimize operative morbidity, we did not perform a formal lymph node dissection.

High risk malignant GISTs can lead to local recurrence. A rupture of the tumor has to be strictly avoided during surgery to prevent intraperitoneal seeding and hemorrhage. A gentle touch technique should be applied, as GISTs tend to have a friable consistency.

Several surgical techniques have to be discussed for the therapy of duodenal GIST. Small tumors might be treated by local excision and primary closure of the duodenal wall, if the remaining lumen is adequate. Segmental resection of the duodenum with the need of a duodenojejunostomy, as performed in our patient, is another possibility. Finally, especially tumors located near to the ampulla of Vater may lead to a duodenopancreatectomy. In the series of Miettinen, about 20% of patients underwent duodenopancreatectomy, whereas segmental resection and local wedge resection were performed in 45% and 20%, respectively [2]. Uehara reports an even higher proportion of 40% of patients treated by duodenopancreatectomy for duodenal GIST [29]. As only 30% of duodenal GISTs show a malignant appearance, patients might in part be over treated by duodenopancreatectomy, especially as this procedure leads to a significant morbidity and mortality.

Only little evidence is available on the choice of surgical procedures for duodenal GIST. For small tumors, local wedge resection of the duodenum is feasible. For smaller gastric GISTs, wedge resection instead of gastrectomy seems to be oncologically adequate. It is unclear if this also true for duodenal GISTs, as Aparicio [23] reported that the risk of local recurrence was higher following peritumoral resection as compared to segmental organ resection. Although an adequate width of tumor-free resection margin has not been defined yet, we hypothesize that a segmental duodenal resection is oncologically superior to a local wedge resection. In a series of 14 patients with duodenal GIST, Goh could show that segmental duodenal resection and duodenopancreatectomy resulted in comparable disease free survival. No local recurrences were observed in both groups [6].

Taken together, existing data suggest that segmental duodenal resection offers equal oncological results as duodenopancreatectomy. As duodenopancreatectomy leads to a significant morbidity and mortality, especially when dealing with a soft pancreatic stump with a narrow pancreatic duct, we advocate segmental duodenal resection as performed in our patient.

Even tumors located very close to the ampulla do not necessitate a duodenopancreatectomy, if they can be resected with an anastomosis just below the ampulla, as we did in the presented case. In order to avoid a stricture of the ampulla, we decided to do a lateroterminal anastomosis located opposite to the papilla. Other authors performed a papilloplasty and inserted a temporary stenting catheter into the papilla to avoid stenosis following a anastomosis located very close to the papilla [26]. Even periampullary GISTs do not have to trigger duodenopancreatectomy. Cavallini reported the case of a periampullary GIST with a diameter of 3.5 cm that was treated by local excision and duodenal wall defect repair, preferring this more conservative procedure to a duodenopancreatectomy. The patient was free of recurrence 4 years after surgery [20]. This underlines that we have to question the indication for duodenopancreatectomies for a tumor with potentially benign appearance. However, tumors of the duodenal bulb, and large tumors with high malignant potential, or tumors reaching into adjacent organs still make a duodenopancreatectomy necessary.

Until recently, the outcome of recurrent and metastatic GIST was fatal, as response rates to conventional chemotherapy were < 5%. Imatinib (Gleevec, Novartis, Basel, Switzerland) is a recently developed selective inhibitor of several tyrosine kinases including KIT. This drug leads to a partial response in 65–70% of patients, while 15–20% reach a stable disease [1,30]. Neoadjuvant or adjuvant use of Imatinib is presently evaluated under study conditions. As our patient was classified as "low risk", we did not initiate an adjuvant treatment with Imatinib.

## Conclusion

We present a case of a duodenal GIST located 3 cm distal of the ampulla of Vater successfully treated by a segmental duodenal resection. We advocate segmental duodenal resection instead of duodenopancreatectomy, as existing data show that even tumors close to the ampulla of Vater may be effectively and safely treated by partial resection of the duodenum, avoiding the higher morbidity and mortality of a duodenopancreatectomy.

## Competing interests

The authors declare that they have no competing interests.

## Authors' contributions

RM reviewed the presented patient's file, prepared images and photographs, performed the review of the literature, and drafted the manuscript. HHW participated in the review of the literature, and helped with the draft of the manuscript. BS contributed all aspects of histology and pathology, prepared the histological photographs, and reviewed the manuscript. FWP conceived of the study, participated in preparing the case report, and supervised the review of the literature. All authors read and approved the final manuscript.

## Consent

Written informed consent was obtained from the patient for publication of this case report and any accompanying images. A copy of the written consent is available for review by the Editor-in-Chief of this journal.
